# Quantum Chemical Density Matrix Renormalization Group Method Boosted by Machine Learning

**DOI:** 10.1021/acs.jpclett.5c00207

**Published:** 2025-03-24

**Authors:** Pavlo Golub, Chao Yang, Vojtěch Vlček, Libor Veis

**Affiliations:** † J. Heyrovsky Institute of Physical Chemistry, v.v.i., Czech Academy of Sciences, Prague, 18223, Czech Republic; ‡ Applied Mathematics and Computational Research Division, Lawerence Berkeley National Laboratory, Berkeley, 94720, United States; § Department of Chemistry and Biochemistry, University of California, Santa Barbara, Santa Barbara, 93117, United States; ∥ Department of Materials, University of California, Santa Barbara, Santa Barbara, 93117, United States; ⊥ J. Heyrovský Institute of Physical Chemistry, v.v.i., Czech Academy of Sciences, Prague, 18223, Czech Republic

## Abstract

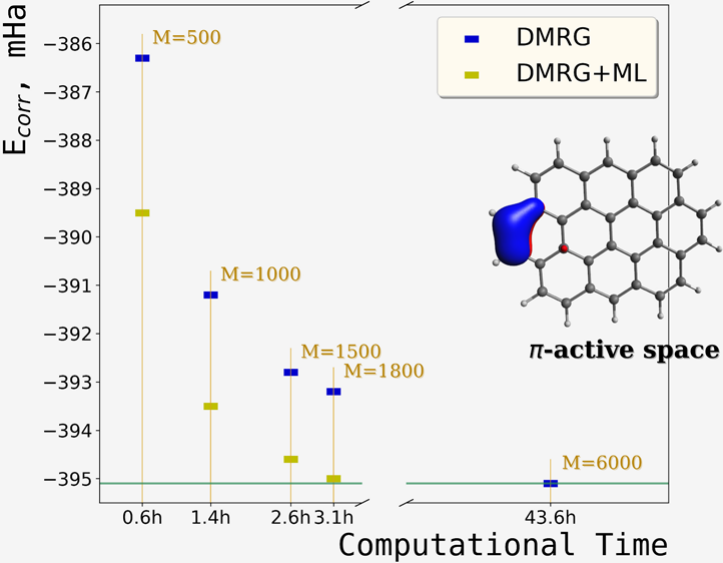

The use of machine learning (ML) to refine low-level theoretical calculations to achieve higher accuracy is a promising and actively evolving approach known as Δ-ML. The density matrix renormalization group (DMRG) is a powerful variational approach widely used for studying strongly correlated quantum systems. High computational efficiency can be achieved without compromising accuracy. Here, we demonstrate the potential of a simple ML model to significantly enhance the performance of the quantum chemical DMRG method.

The concept of using machine learning (ML) to refine low-level theoretical calculations, bringing them closer to high-level accuracy, is a promising and actively evolving approach known as Δ-ML.^1^ Various computational starting points have been explored in this context, including density functional theory (DFT)^2−7^ or Hartree–Fock (HF) single-reference calculations,^8^ and post-HF *ab initio* methods like second order Møller–Plesset perturbation thoery (MP2)^9,10^ or coupled clusters with singles and doubles (CCSD).^11^ Additionally, variational two-electron reduced-density matrix (v2RDM) descriptions have been used as starting points.^12^ These methods are optimized against highly accurate, yet computationally intensive, benchmarks such as coupled clusters with perturbative triples [CCSD(T)] or complete active space configuration interaction (CASCI), aiming to achieve results that closely approximate high-level accuracy.

The DMRG method^13,14^ is a powerful variational approach widely used for studying strongly correlated quantum systems.^15^ By optimizing the many-body wave function within a truncated Hilbert space, DMRG achieves high computational efficiency without compromising accuracy. In quantum chemistry applications,^16−20^ DMRG typically approximates the ground state (or low-lying excited states) of a full configuration interaction (FCI) solution within a chosen orbital space, such as that defined by the CASCI framework. An example can be the π-orbital active space of polycyclic aromatic hydrocarbons (PAHs) presented below.

The DMRG algorithm provides the wave function in a matrix product state (MPS) representation, which allows for an efficient and compact description of entangled quantum states.^21^ The FCI wave function, in the occupation basis representation, is expressed as
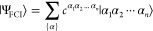
1where α_*i*_ represents the occupation state of the *i*-th orbital, with α_*i*_ ∈ |0 ⟩, |↓⟩, |↑⟩, |↓↑⟩. By successively applying singular value decomposition (SVD) to the FCI tensor *c*^α^_1_ α_2_... α_*n*_, the wave function can be factorized into an MPS form^21^
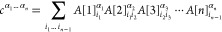
2where *A*[*j*]^α_*j*_^ are the MPS matrices corresponding to each orbital. The new indices, *i*_*j*_, introduced by SVD, are called virtual indices, and they are contracted across different MPS matrices. If the MPS factorization was exact, the dimensions of these matrices would grow exponentially with a system size, similar to the growth of the original FCI tensor. In the DMRG algorithm, however, the dimensions of the virtual indices are truncated, resulting in the reduced computational complexity. They are called bond dimensions and are typically denoted by *M*. The choice of *M* controls the accuracy of the approximation, with larger bond dimensions capturing more entanglement at the cost of higher computational demands.

The iterative protocol of the practical two-site DMRG algorithm assumes that the orbitals are arranged in a 1D chain. The system is divided into two large blocks (left and right) with two smaller blocks, each consisting of a single orbital, positioned between them. The algorithm performs a sweeping process from left to right, gradually enlarging the left block by one orbital while shrinking the right block by the same amount. Once the end of the chain is reached, the sweep reverses direction. In each iteration of the sweep, the eigenvalue problem corresponding to the projected Schrödinger equation onto the tensor product space of the aforementioned four blocks is solved.

In the original DMRG formulation,^13−15^ the explicit determinant representations of the complex many-particle bases are not stored. Instead, the matrix representations of second-quantized operators required for applying the Hamiltonian to a (trial) wave function are constructed and retained. Transitioning between iterations during a DMRG sweep occurs via a renormalization procedure. A key component of the DMRG algorithm is truncation, achieved through SVD of the wave function in its bipartite form, expanded in the basis of the enlarged left and right blocks
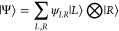
3| *L*⟩ and |*R*⟩ represent the basis states of the merged left and right blocks, each including a neighboring single orbital. The 4*M* × 4*M* matrix ψ_*LR*_ is approximated by *M* × *M* matrix ψ̃_*LR*_ by using only the largest singluar values. Alternatively, this truncation can be achieved by diagonalizing the density matrix of the enlarged left or right block and preserving only the *M* largest eigenvalues. A key indicator of the accuracy of the approximation at a particular iteration of the DMRG sweep is the truncation error (TRE), given by
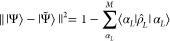
4where ρ̂_*L*_ represents the reduced density operator of the enlarged block and |α_*L*_⟩ denote its eigenvectors.

An important characteristic of the quantum system under study, easily accessible through the DMRG calculation, is its entanglement properties, which can be quantified using the *N*-orbital entanglement entropy as defined by the von Neumann entropy.^22−24^
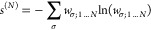
5where *w*_σ;1,···*N*_ represent the eigenvalues of the reduced *N*-orbital density matrix. For example a single-orbital entropy, *s*_*i*_^(1)^, quantifies the entanglement between a single orbital *i* and the remaining subset of orbitals, while the two-orbital entropy, *s*_*ij*_^(2)^, measures the entanglement between a pair of orbitals *i* and *j* and the rest of the system. The mutual information, which reflects the correlation between a specific pair of orbitals, is given by the following expression
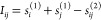
6As mentioned above, TRE quantifies the accuracy of the wave function. It has been observed empirically^25−27^ that the maximum TRE from the last sweep before the convergence is achieved (also denoted as discarded weight) is almost linearly proportional to the error in the DMRG energy. This fact allows for extrapolations to the truncation error zero limit.^27^ Accurate extrapolations, however, require multiple calculations with increasing bond dimensions spanning several orders of magnitude in TRE. Given the scaling of the DMRG algorithm with bond dimension, , it is evident that this process can become computationally expensive.

In this letter, we introduce an alternative approach. We assume that single-orbital entropies and mutual information capture sufficient information to predict the behavior of the DMRG energy error as the bond dimension increases. In the spirit of Δ-ML methods, we propose using correlation measures from calculations with significantly lower bond dimensions to estimate energies in the zero truncation error limit. Since the dependence patterns may vary across different types of correlated systems and are often complex, nonobvious, or nonuniform machine learning techniques are particularly well-suited to uncover and model these hidden relationships.

A way to unify the analysis of molecules of varying sizes is by representing them as graph data structures. This involves organizing the available information so that part of the data corresponds to relatively separable entities (nodes), while the remaining data is associated with pairs of nodes (edges). For instance, a natural way to represent real-space molecular structures as graphs is by treating constituent atoms as nodes and atomic bonds as edges. In machine learning, the processing of graph-structured data falls under the domain of graph neural networks (GNN).^28,29^

In quantum chemistry, DMRG is typically applied to a set of molecular orbitals. In this context, a natural way to represent the system as a graph is to treat each individual orbital as a node. Consequently, single-site entropies become node features, while mutual information (or two-site entropies) can serve as edge features. In this work, we used the mutual information value as the edge feature and set a minimal threshold of 0.004 for edge existence. Additionally, we incorporated the DFT orbital occupancy information on a pair of orbitals as another edge feature (see Supporting Information, SI, for more details). Alternative option would be to use occupancy information as a node feature, which however results in more learning parameters as discussed in Supporting Information.

In our proof-of-concept study, we employ a simple message-passing graph neural network (MPGNN) approach, as illustrated in Figure 1. The graph representation is constructed based on mutual information, using a predefined threshold (Step II in Figure 1). In the next stage (Step III), messages are formed by concatenating the node and edge features of connected neighbors. These messages are then processed by a differentiable function *f*, such as a deep neural network. Step IV in Figure 1 illustrates the message update process, which involves aggregating messages from connected neighbors, concatenating the aggregated messages with the node features, and processing them with another differentiable function *g*. The aggregation operation, denoted by ⊕, can use various pooling techniques, such as summation, mean, or others; in this study, we use mean pooling.

**Figure 1:**
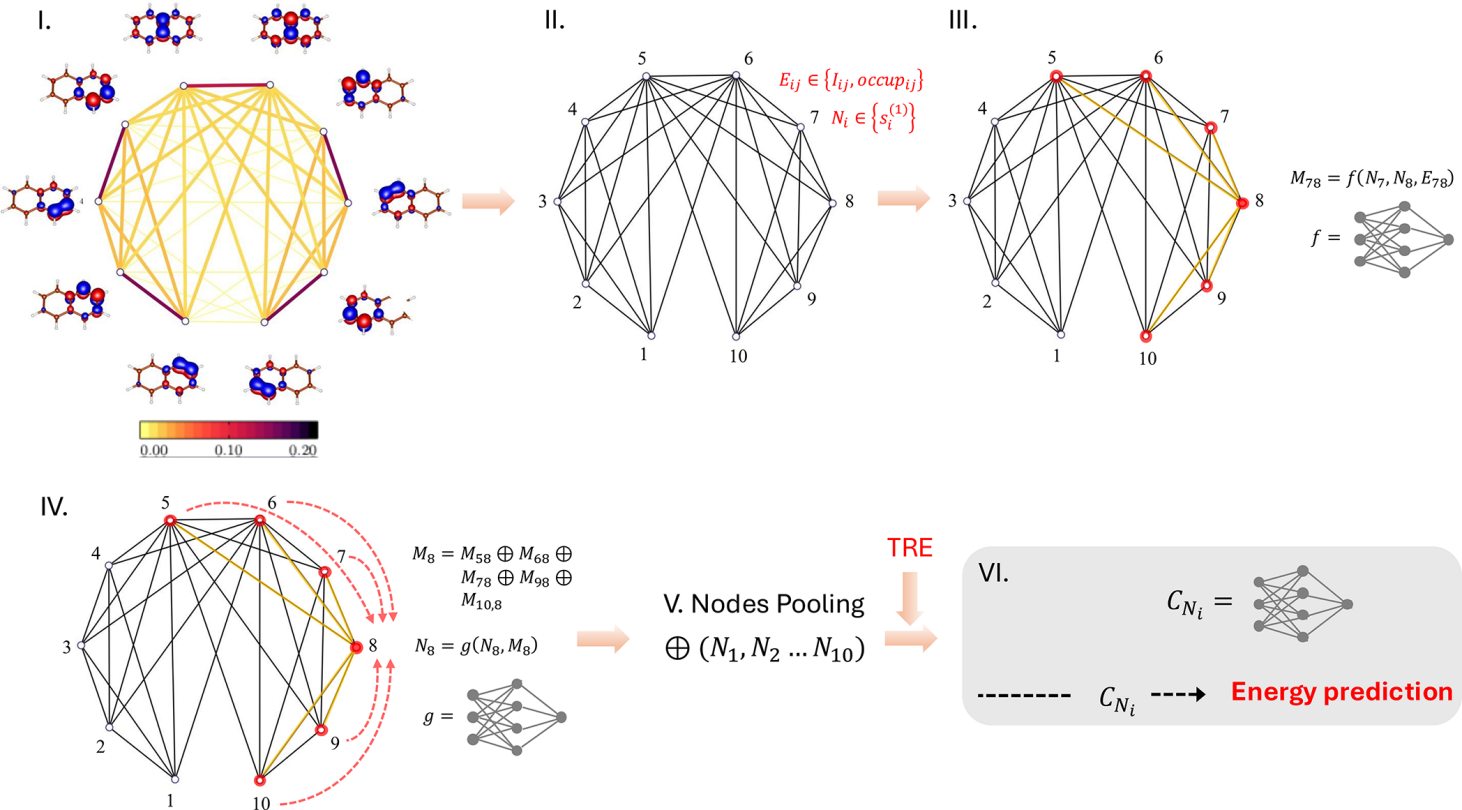
Schematic representation of the MPGNN model used in this work. (I) Mutual information plot. (II) Corresponding graph representation, showing node and edge features. (III) Message formation comprising neural network processing (*f*). (IV) Message update comprising message pooling and neural network processing (*g*). Steps III and IV are repeated *k*-times for a *k*-layer MPGNN. (V) Final pooling of node representations. (VI) Neural network-based energy prediction. For further details, refer to the main text.

After *k*-layer message passing, the graph-level representation is constructed by applying an aggregation operator across all nodes. This graph representation is then augmented with the corresponding truncation errors and fed into a fully connected neural network, *C*, which outputs the energy prediction. The learning target was the difference between the reference high-bond-dimension DMRG energy and current low-bond-dimension DMRG energy divided by the absolute value of the uncorrelated energy. Such a normalization has been applied to avoid learning on absolute energies that are size-dependent and to work instead with fractions.

In this study, we utilized the simplest form of an MPGNN, consisting of a single message-passing layer, with *f* and *g* represented as identity functions. This basic MPGNN configuration is the primary focus throughout the main text. Results for more complex MPGNN architectures, along with a detailed description of both network configurations, are provided in Supporting Information.

The training data set consisted of elements from the publicly available database of polycyclic aromatic hydrocarbons (PAHs) COMPAS-1D.^30^ It included 100 molecules: all PAHs with 5 and 6 benzene rings (49 molecules in total) and selected 51 PAHs with 7 benzene rings and the smallest HOMO–LUMO gaps.

The test data set was intentionally designed to include peri-fused PAHs that are absent from the COMPAS-1D database. The selected molecules are shown in Figure 2. It is well-known that the electronic structure of PAHs depends heavily on molecular topology.^31,32^ In order to demonstrate that our ML model is agnostic to this property, we also included [3]triangulene (system I), which unlike molecules in the training data set exhibits sublattice imbalance, resulting in a triplet ground state. To evaluate performance in the presence of heteroatoms, the data set included the aza-analogue of [4]triangulene (system V). Furthermore, to test the model’s transferability to more extended systems, we included peripentacene (system IV), the largest molecule in the test data set (14 benzene rings), which is considerably larger than molecules in the training data set. In addition, peripentacene exhibits the open-shell singlet ground state and extrapolated DMRG reference singlet–triplet (S-T) energy gap is available.^33^

**Figure 2:**
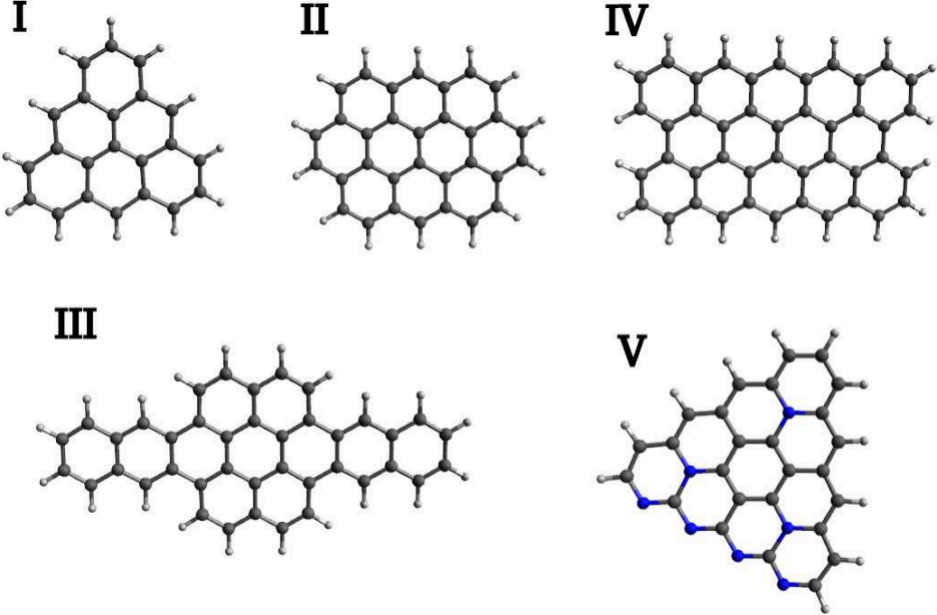
Test set of polycyclic aromatic hydrocarbons: **I** – C_22_H_12_, [3]triangulene; **II** – C_32_H_14_, ovalene; **III** – C_40_H_20_; **IV** – C_44_H_18_, peripentacene; **V** – C_26_N_7_H_11_.

The geometries of the molecules in the training data set were obtained from the COMPAS-1D database,^30^ while those in the test data set were adopted from other studies and are provided in Supporting Information. Input orbitals for DMRG calculations were computed at the DFT level using the B3LYP exchange-correlation functional^34,35^ and the cc-PVDZ basis set.^36^

It is well established that DMRG achieves optimal performance in a local basis,^27^ where the MPS parametrization effectively leverages the locality of electron correlation. To ensure this, the initial DFT orbitals were split-localized using the Pipek-Mezey procedure.^37^ All calculations for PAHs presented in this study employed complete π-active spaces, corresponding to CAS sizes of (22e, 22o), (26e, 26o), and (30e, 30o) for molecules containing 5, 6, and 7 benzene rings, respectively. For each molecule, three DMRG calculations were performed at low bond dimensions, resulting in truncation errors ranging from 1 × 10^–4^ to 5 × 10^–6^. High-accuracy reference calculations achieved truncation errors as low as 10^–7^ or better.

For molecules in the test data set, the active spaces were reconstructed by selecting *p*_*z*_ orbitals of carbon and nitrogen atoms following the split-localization procedure. This process yielded the following CAS configurations: I – (22e, 22o), II – (32e, 32o), III – (40e, 40o), IV – (44e, 44o), and V – (36e, 33o).

The results of our Δ-ML DMRG approach, compared to standard DMRG, for the lowest singlet state energies of PAHs **I**, **II**, **III**, and **V** from the test set are shown in Figure 3. As illustrated, the ML-corrected DMRG consistently provides lower (and occasionally very slightly overshot) energies that are more accurate than the standard DMRG.

**Figure 3:**
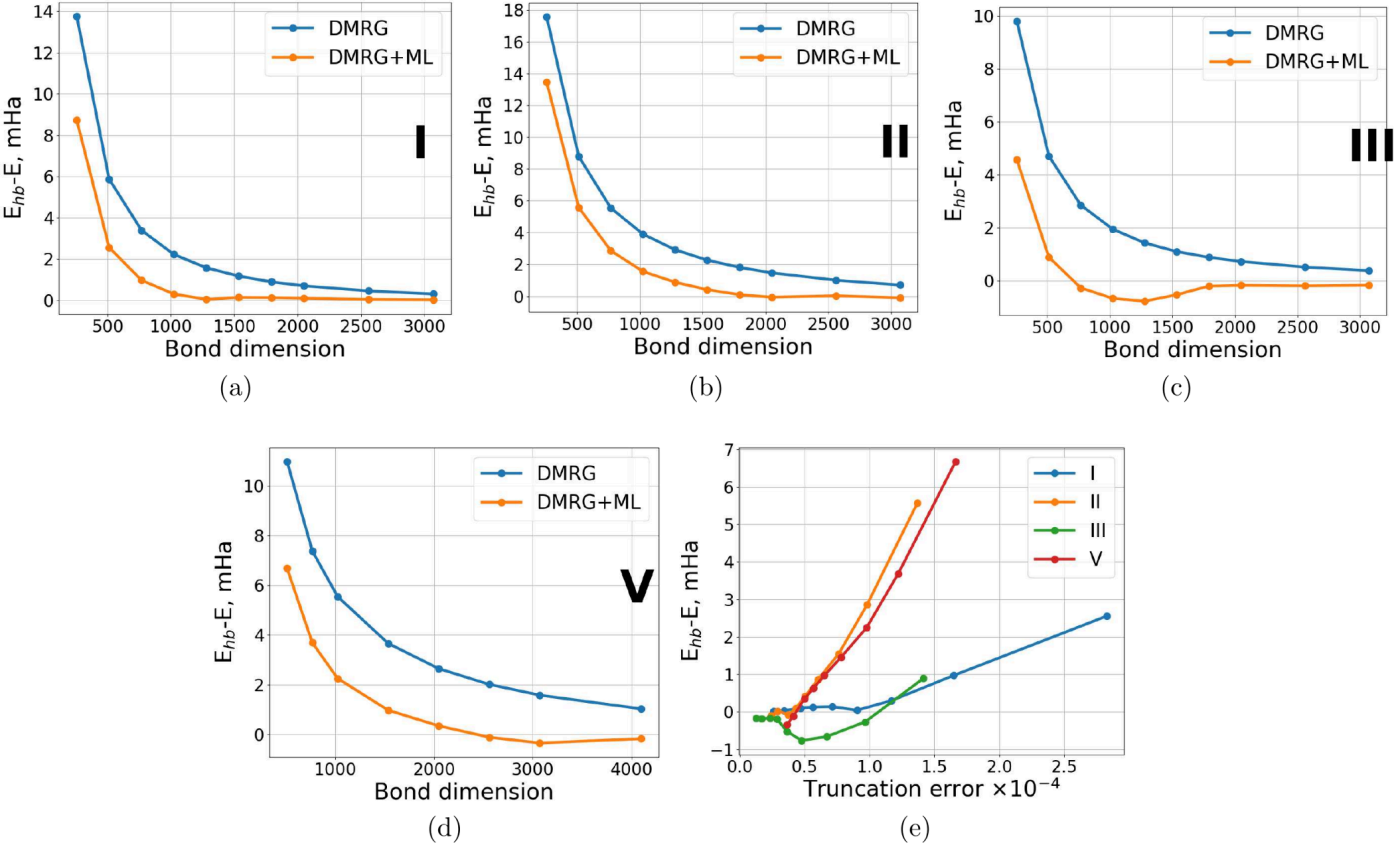
(a–d) Differences between reference (high-bond-dimension, *E*_*hb*_) DMRG energies and standard DMRG energies, as well as ML-corrected DMRG energies, calculated across different bond dimensions for the singlet states of molecules shown in Figure 2. (e) Dependence of DMRG+ML energies on the truncation error of the parent low-bond-dimension DMRG calculations for all four molecular examples.

At very low bond dimensions, the ML corrections do not yet achieve chemical accuracy (1 kcal/mol). At these bond dimensions, the results are insufficiently accurate to serve as reliable input for the model. However, as the bond dimension increases, the quality of the ML corrections improves. For bond dimensions between 750 and 1250, the difference between the ML-corrected energies and *E*_*hb*_ energies consistently falls below 1 mHa, as seen for all test molecules. The performance of the ML model is further validated as the bond dimension approaches the high-quality limit, where the ML corrections maintain accuracy without overestimating the energies.

Figure 3e shows that, at TRE of 5 × 10^–5^ (system **II**), the accuracy of the ML corrections reaches 1 mHa in the worst case. For the other examples, this level of accuracy is achieved at higher truncation errors.

A particularly challenging test for the ML model is to correctly predict energy gaps between close-lying electronic states. Depending on their size, geometry, and edge structure, peri-fused PAHs can adopt either closed-shell singlet, open-shell singlet, or higher-spin ground states, with the energy difference between them often being very small. Diradical neutral [3]trianguelene (**I**) is known to be the smallest PAH with triplet ground state. Owing to the maximum overlap of the π-radical wave functions it is considered to have the largest singlet–triplet (S-T) gap among all PAH diradicals.^31^ In contrast, peripentacene (**IV**) exhibits a singlet open-shell ground state. Its S-T gap has been estimated to be around 130 meV from all-π DMRG calculations, and approximately 49 meV after accounting for the substrate effects and dynamical correlation corrections via multireference-coupled cluster methods.^33^

In Figure 4, we present the performance of the ML corrected DMRG on the S-T gap of [3]triangulene. It is evident that our Δ-ML DMRG approach correctly predicts the S-T gap value already at M = 750, which corresponds to TRE in the range of 1–3 × 10^–4^. A slight deviation is observed with increase of the bond dimension, however, this deviation never exceeds 1 mHa in absolute value.

**Figure 4:**
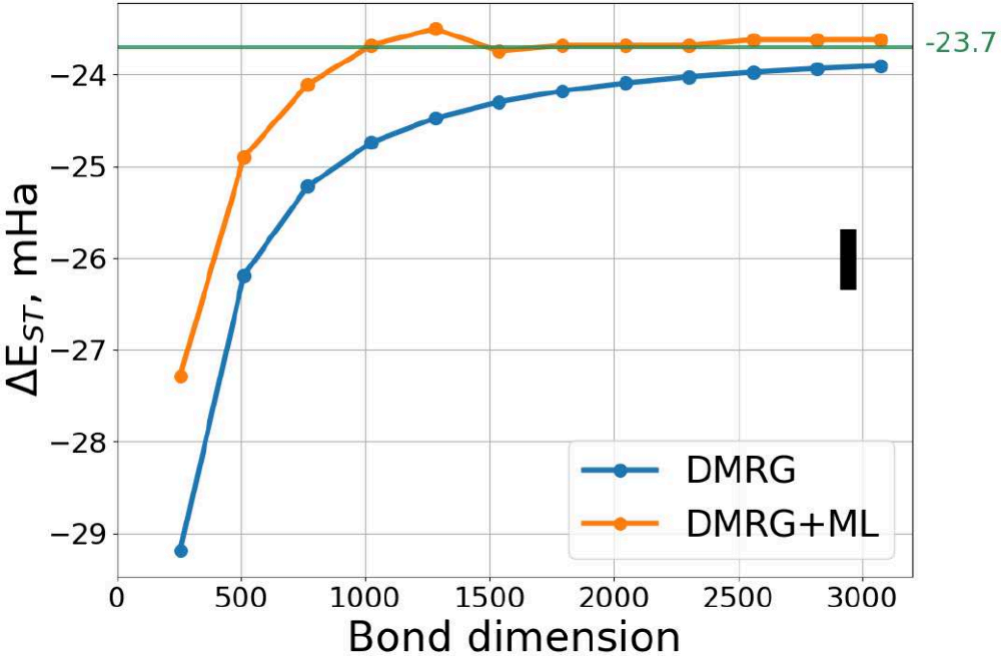
Singlet–triplet (S-T) gap for [3]triangulene (**I**) computed at various bond dimensions. The reference S-T gap value of −2.375 × 10^–2^ Ha is obtained from a DMRG calculation with a bond dimension of 5000.

Figure 5 illustrates the S-T gap results for peripentacene. In this case, standard DMRG even fails to predict the correct ground state at low bond dimensions. In contrast, the Δ-ML DMRG method predicts the correct order of both electronic states much earlier, already at M = 500 and TRE of 1 × 10^–4^. For bond dimensions greater than 500, Δ-ML DMRG shows stable predictions within the range 4.5–4.7 × 10^–3^ Ha, closely aligning with the reference value of 4.78 × 10^–3^ Ha, obtained by Sánchez-Grande et al.^33^ after extrapolation of DMRG energies at high bond dimensions with respect to TRE. This remarkable agreement is particularly promising, especially considering that, despite selecting PAHs from the COMPAS-1D database with the smallest HOMO–LUMO gaps, none of the systems in the training set exhibited an open-shell singlet ground state like that of peripentacene.

**Figure 5:**
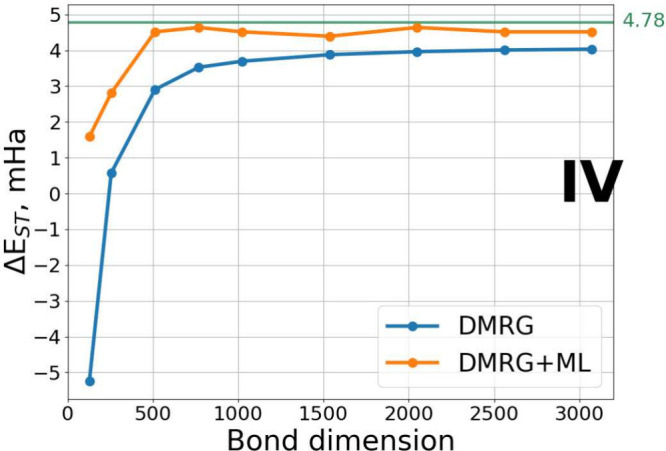
Singlet–triplet (S-T) gap for peripentacene (**IV**) computed at various bond dimensions. The reference S-T gap value of 4.78 × 10^–3^ Ha is taken from Sánchez-Grande et al.^33^

Unlike the extrapolation scheme used to estimate the zero truncation error limit, the presented ML model requires only a single input point, whereas extrapolation demands multiple data points. Moreover, the extrapolation scheme performs better when the extrapolated points are closer to the exact energies. For example, it makes qualitatively accurate extrapolations in the case of [3]triangulene. However, in more complex cases that require significantly higher bond dimensions, such as peripentacene, its accuracy declines when using the same low-bond dimensions (see Figure S4 and Figure S5 in Supporting Information). Regarding the potential application of the current ML model for predicting excited-state energies, its accuracy depends on state-specific truncation errors. If only state-averaged truncation errors are available, its performance may be compromised.

In summary, we demonstrated the potential of a simple ML model to significantly enhance the performance of the quantum chemical DMRG method. The model leverages minimal input from low-cost DMRG calculations – such as single- and two-site entropies, truncation error, and orbital occupancies – without requiring prior knowledge of molecular geometry or other properties. This approach substantially improves the accuracy of low-cost DMRG energies and energy gaps. We applied the model to the electronic structure of polycyclic aromatic hydrocarbons, making the trained model, available online,^38^ directly applicable to general organic aromatic π-extended systems, including aromatic heterocycles.

Furthermore, we believe similar models can be developed for other classes of strongly correlated molecules, such as transition metal complexes.^39^ To support this, we advocate for the systematic tabulation and sharing of DMRG calculation results.

## Supporting Information Available

Computational and ML model details, Geometries, Additional results and References, Tables with geometry of system in XYZ format, Figures showing plotted data resulting from calculations (PDF)
Additional plots of data resulting from calculations (ZIP)
