# Supplementary material for: Quantum Chemical Density Matrix Renormalization Group Method Boosted by Machine Learning
Source: J Phys Chem Lett. 2025 Mar 24;16(13):3295–301. doi: 10.1021/acs.jpclett.5c00207 (PMC11973911; doi:10.1021/acs.jpclett.5c00207)
Supplement: Supplementary file 1 — jz5c00207_si_001.pdf [file jz5c00207_si_001.pdf]

Supporting Information: Quantum Chemical Density Matrix Renormalization Group Method Boosted by Machine Learning

Pavlo Golub,*,† Chao Yang,‡ Vojtěch Vlček,¶ and Libor Veis*,§

†*J. Heyrovsky Institute of Physical Chemistry, v.v.i., Czech Academy of Sciences, Prague, Czech Republic*

‡*Applied Mathematics and Computational Research Division, Lawerence Berkeley National Laboratory, Berkeley, USA, 94720.*

¶*Department of Chemistry and Biochemistry, University of California, Santa Barbara, Santa Barbara, USA, 93117 and Department of Materials, University of California, Santa Barbara, Santa Barbara, USA, 93117.*

§*J. Heyrovský Institute of Physical Chemistry, v.v.i., Czech Academy of Sciences, Prague, Czech Republic.*

E-mail: pavlo.golub@jh-inst.cas.cz; libor.veis@jh-inst.cas.cz

Computational and ML model details

Input orbitals for DMRG calculations were computed at the DFT level using the B3LYP exchange-correlation functional[S1,S2] and the cc-PVDZ basis set.[S3] To improve the performance of DMRG and facilitate active space selection the split-localization procedure (separate localization of occupied and virtual orbitals) by means of the Pipek-Mezey method[S4]

was used. Complete π-active spaces were selected afterward, leading to the following active space sizes – (22e, 22o), (26e, 26o), (30e, 30o) for molecules with 5, 6, and 7 benzene rings (5C, 6C, and 7C) respectively. The ordering of the active orbitals mapped onto the 1D lattice of DMRG sites was obtained with the Fiedler method[S5] applied on the matrix of exchange integrals.[S6] All DMRG calculations were initialized with the CI-DEAS procedure.[S7]

DMRG calculations with bond dimensions 64, 128, and 256 for 5C and 6C molecules, and with bond dimensions 128, 256, and 512 for 7C molecules were used as training points. This ensured the input data with the truncation error in the 1×10^{-4} – 5×10^{-6} range. The reference targets were obtained with bond dimension 3000 for 5C and 6C molecules, and with bond dimension 4000 for 7C molecules. The reference accurate calculations ensured the truncation error ranged from 10^{-7} to lower orders.

Test molecules were subject to the same active space selection procedure, that resulted in the following active space configurations: **I** – (22e, 22o), **II** – (32e, 32o), **III** – (40e, 40o), **IV** – (44e, 44o), **V** – (36e, 33o).

Message passing graph neural network (MPGNN) was chosen as a machine learning model. Within the MPGNN framework for each node an embedding is built based on its connections. To briefly introduce the architecture of the MPGNN employed in this work, consider node 1 with node features N_1^{k-1} from $(k-1)$th massage passing layer, and connected to nodes 2 and 3 with node features N_2^{k-1}, N_3^{k-1} respectively, and edge features E_{12}, E_{13}. Initially, messages from the connected neighbors are generated by concatenating their node and edge features and processing them using a differentiable function f (e.g. fully connected neural network)

$$M_{12}^k = f(N_1^{k-1}, N_2^{k-1}, E_{12}), \quad (1)$$

$$M_{13}^k = f(N_1^{k-1}, N_3^{k-1}, E_{13}). \quad (2)$$

Next, the messages from the connected neighbors are aggregated using an operation $\bigoplus$ (e.g., summation or mean) as $\bigoplus\left(M_{12}^k, M_{13}^k\right)$ and concatenated with the node features from the $(k-1)$th massage passing layer, N_1^{k-1}. This combined vector is then passed through another set of fully connected neural network layers (referred to as the message update), function g, resulting in updated node features N_1^k for the kth massage passing layer. By applying multiple message passing layers, feature representations for all nodes are obtained, capturing their specific interconnections. Finally, a feature representation of the entire graph can be constructed by applying an aggregation function to the final feature vectors of all graph nodes.

The simplest model (**model1**), whose results are presented in the main text, assumed one message passing layer with f and g being identity functions. The second model (**model2**), whose results are presented below, featured three message passing layers. In this model, the function f and g were single-layer fully connected neural networks with 8 neurons each, using ReLU activation ($f(z) = max\{0, z\}$) for each neuron. Both models featured mean aggregation function. Resulted graph representation was processed through tree-layer fully-connected neural network, with 20 neurons in each layer and leaky ReLU ($f(z) = max\{0, z\} + 0.2 \cdot min\{0, z\}$) activation function on each neuron. To avoid overfitting dropout regularization[S8,S9] procedure was used with dropout rate 0.1. Each train procedure run over 1500 epochs.

One-site entropy was used as a node feature, while mutual information served as an edge feature. Additionally, orbital occupation information, encoded as a three-element one-hot vector (*occup*), was incorporated into the edge features. If both nodes were associated with occupied orbitals, then $occup[0] = 1$. If both were associated with virtual orbitals, $occup[2] = 1$. Otherwise, $occup[1] = 1$. In other words

$$occup = \begin{cases} [1\ 0\ 0] & \text{if both orbitals are occupied,} \\ [0\ 1\ 0] & \text{if one orbital is occupied, and another is virtual,} \\ [0\ 0\ 1] & \text{if both orbotal are virtual.} \end{cases} \quad (3)$$

Alternatively, occupancy information can be used as a node feature, represented as a two-element one-hot vector (occcup[0]=1 if the orbital is occupied, and occcup[1]=1 if the orbital is virtual) at each node. However, in this case during message formation the two-element one-hot vectors from connected nodes are concatenated, resulting in four input learning parameters in total, which is one more in comparison to the case, when occupation information is encoded as edge feature.

TensorFlow machine learning platform[S10] and PyTorch Geometric library[S11] were used to built the models.

Additional results

Model2 (Figure S1–S3) exhibits trends similar to **model1** discussed in the main text. At bond dimensions ranging from 750 to 1500, the difference between ML corrected energies and E_{hb} energies falls below 1 mHa. The ML correction achieves an accuracy of 1 mHa in the worst case at truncation error of $5{\times}10^{-5}$ (system **II**). However, for the other two examples, this accuracy is achieved already at truncation errors around $1{\times}10^{-4}$ (Figure S1,(e)).

Δ-ML DMRG procedure accurately predicts the S-T gap value for [3]triangulene at $M =$ 1000, Figure S2. For peripentacene (Figure S3), the S-T gap is correctly predicted at $M =$ 500 with a truncation error of $1{\times}10^{-4}$. Across bond dimensions from 500 to 3000, Δ-ML DMRG approach provides stable predictions in the range of 4.8–$5.1{\times}10^{-3}$ Ha.

Final energies and truncation errors of all DMRG calculations performed on systems in training as well as test sets can be found in Ref.[S13]

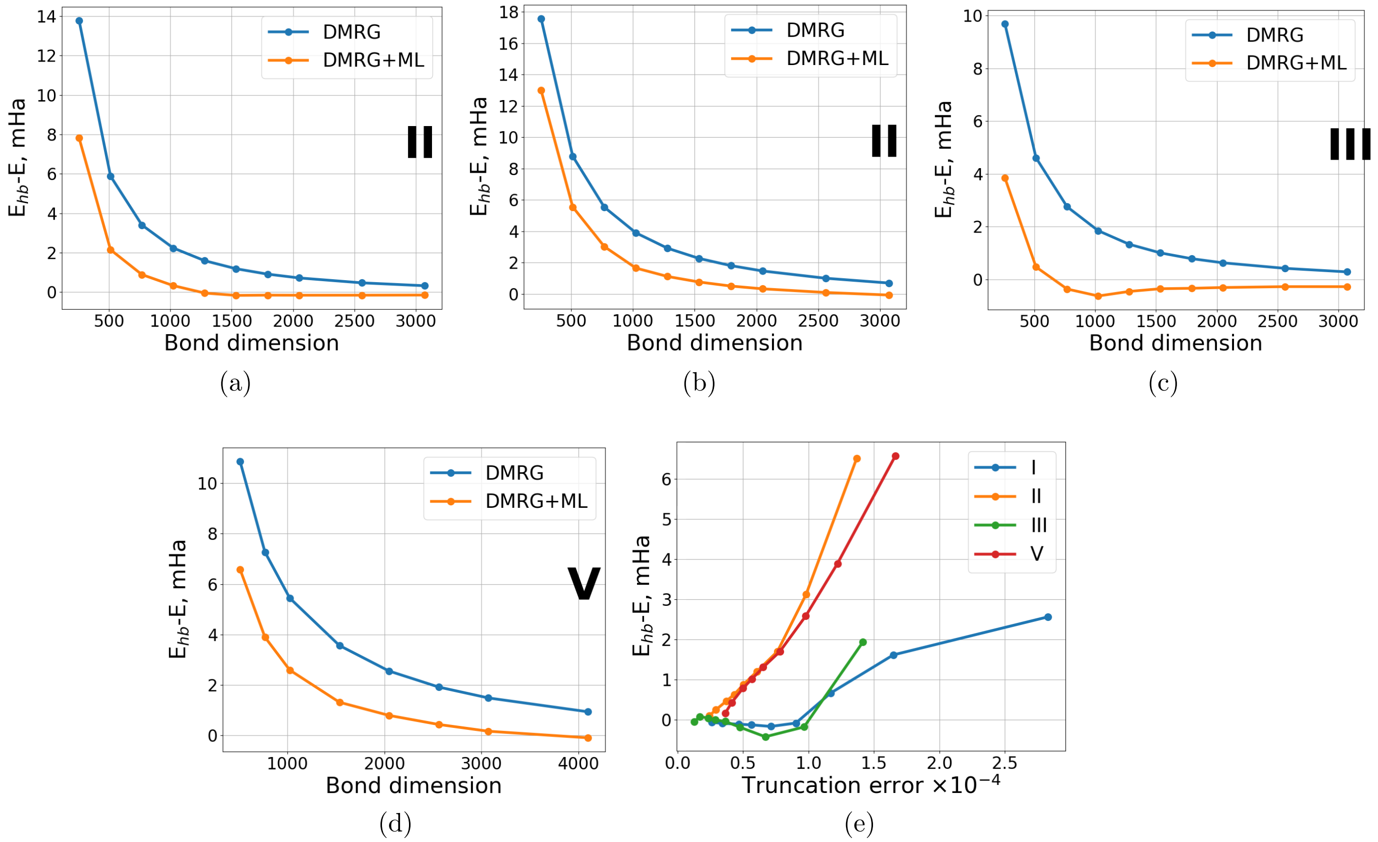

Figure S1: (ad) Differences between reference (high-bond-dimension, E_{hb}) DMRG energies and standard DMRG energies, as well as ML-corrected DMRG energies, calculated across different bond dimensions for the singlet states of molecules shown in Figure 2 of the main text. (e) Dependence of DMRG+ML energies on the truncation error of the parent low-bond-dimension DMRG calculations for all four molecular examples. **model2** predictions.

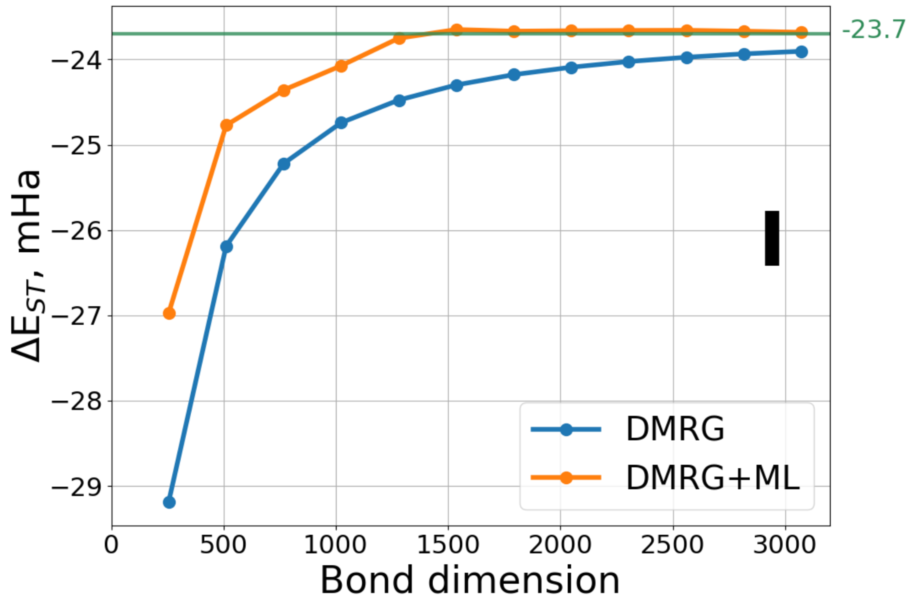

Figure S2: Singlet-triplet (S-T) gap for [3]triangulene (**I**) computed at various bond dimensions. The reference S-T gap value of -2.375×10^{-2} Ha is obtained from a DMRG calculation with a bond dimension of 5000. DMRG+ML uses the predictions of **model2**.

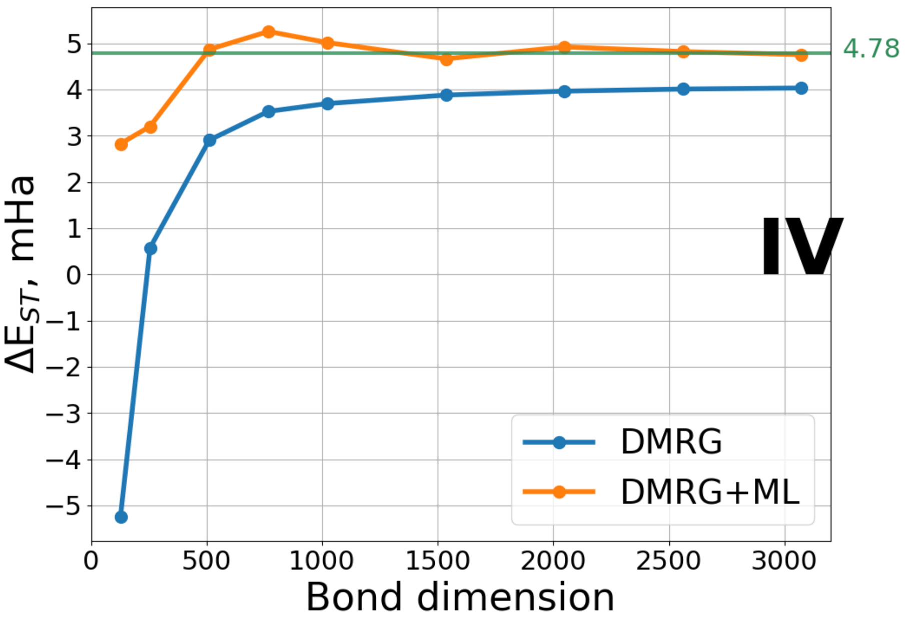

Figure S3: Singlet-triplet (S-T) gap for peripentacene (**IV**) computed at various bond dimensions. The reference S-T gap value of 4.78×10^{-3} Ha is taken from Sánchez-Grande et al.[S12] DMRG+ML uses the predictions of **model2**.

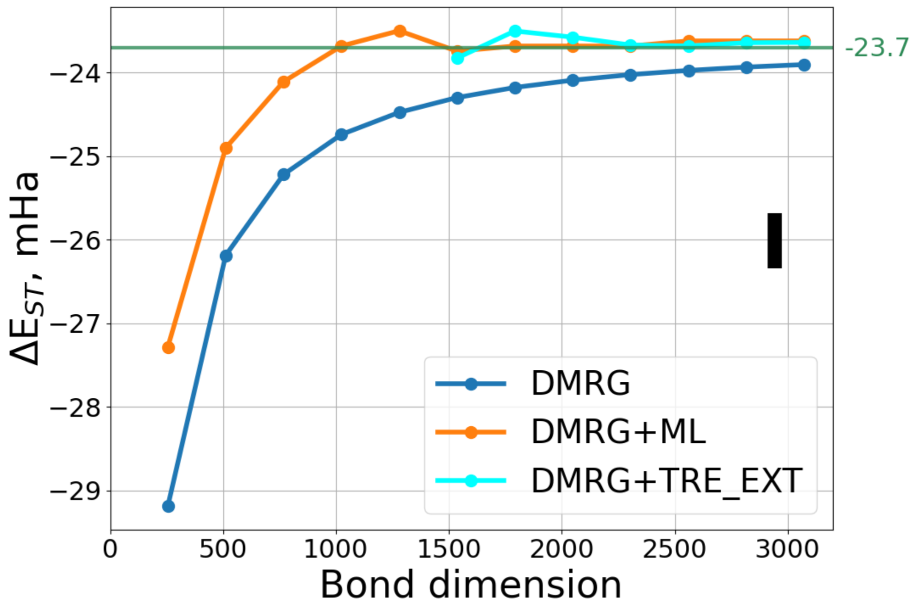

Figure S4: Singlet-triplet (S-T) gap for [3]triangulene (**I**) computed at various bond dimensions. The reference S-T gap value of -2.375$\times 10^{-2}$ Ha is obtained from a DMRG calculation with a bond dimension of 5000. Each point in DMRG+EXT scheme has been obtained by linear extrapolation of the results of the current point and 3 previous points, i.e. the prediction at point 1500 has been obtained by linear extrapolation of energies at bond dimensions 750, 1000, 1250, and 1500; the prediction at point 1750 has been obtained by linear extrapolation of energies at bond dimensions 750, 1000, 1250, and 1500; and so on. Singlet and triplet energies have been extrapolated separately. DMRG+ML uses the predictions of **model1**.

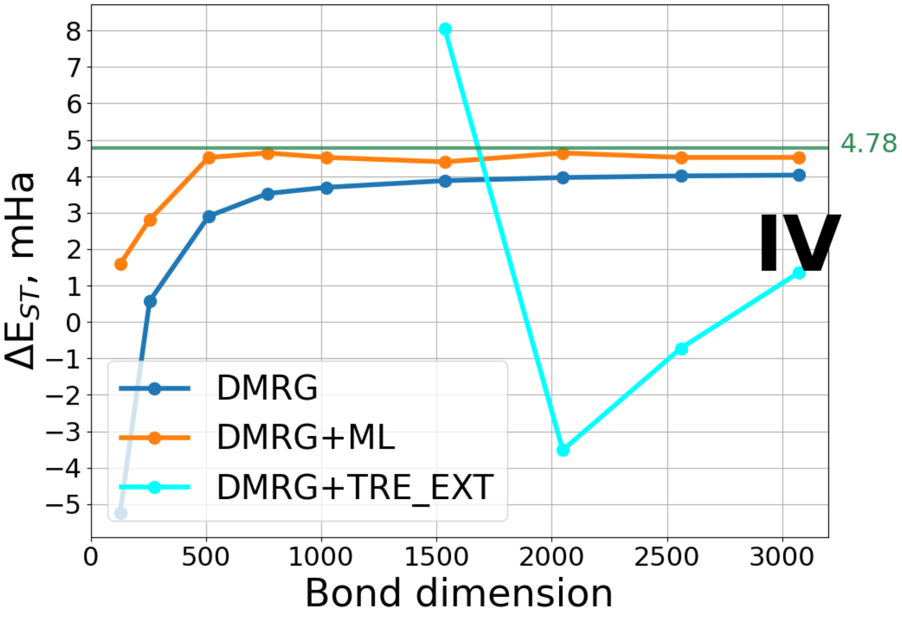

Figure S5: Singlet-triplet (S-T) gap for peripentacene (**IV**) computed at various bond dimensions. The reference S-T gap value of 4.78×10^{-3} Ha is taken from Sánchez-Grande et al.[S12] Each point in DMRG+EXT scheme has been obtained by linear extrapolation of the results of the current point and 3 previous points, i.e. the prediction at point 1500 has been obtained by linear extrapolation of energies at bond dimensions 750, 1000, 1250, and 1500; the prediction at point 1750 has been obtained by linear extrapolation of energies at bond dimensions 750, 1000, 1250, and 1500; and so on. Singlet and triplet energies have been extrapolated separately. DMRG+ML uses the predictions of **model1**.

Geometries

Geometries of the test set molecules are provided in Tables S1 - S5.

Table S1: Geometry of the system **I** ([3]triangulene]) in XYZ format (Å).

C	-4.4227	-0.7180	-0.0001
H	-5.4888	-0.8911	0.0008
C	-3.9178	0.6309	-0.0006
H	-4.6136	1.4569	-0.0003
C	-2.4184	0.9156	-0.0011
C	-1.9322	2.3672	-0.0018
H	-2.6151	3.2039	-0.0025
C	-0.4126	2.5527	-0.0009
C	0.1668	3.9646	-0.0005
H	-0.5030	4.8118	-0.0015
C	1.5895	4.1891	0.0018
H	1.9727	5.1989	0.0034
C	2.5052	3.0773	0.0025
H	3.5685	3.2669	0.0049
C	2.0020	1.6365	0.0005
C	0.5004	1.3190	0.0000
C	-0.0001	-0.0001	-0.0004
C	0.8921	-1.0930	-0.0006
C	2.4170	-0.9189	-0.0010
C	3.3503	-2.1265	-0.0007
H	4.4189	-1.9699	-0.0008
C	2.8334	-3.4709	0.0003
H	3.5164	-4.3076	0.0010
C	1.4127	-3.7082	0.0010
H	1.0454	-4.7238	0.0026
C	0.4162	-2.5522	-0.0007
C	-1.0840	-2.8570	-0.0012
H	-1.4671	-3.8668	-0.0013
C	-2.0045	-1.6338	-0.0009
C	-1.3926	-0.2262	-0.0014
C	-3.5170	-1.8380	0.0052
H	-3.9158	-2.8416	0.0097
C	3.0162	0.4898	-0.0006
H	4.0822	0.6629	-0.0028

Table S2: Geometry of the system **II** (ovalene) in XYZ format (Å).

C	0.0000	-0.7061	-0.0001
C	0.0000	0.7061	-0.0001
C	1.2234	-1.4132	-0.0001
C	1.2233	1.4131	0.0000
C	-1.2234	-1.4132	0.0000
C	-1.2233	1.4132	0.0000
C	2.4487	-0.7068	-0.0002
C	2.4486	0.7068	-0.0001
C	-2.4487	-0.7068	0.0000
C	-2.4486	0.7069	-0.0001
C	1.2227	-2.8249	0.0001
C	1.2228	2.8249	0.0002
C	-1.2228	-2.8249	0.0001
C	-1.2227	2.8249	0.0000
C	3.6724	-1.4131	-0.0002
C	3.6723	1.4130	-0.0001
C	-3.6724	-1.4131	0.0000
C	-3.6723	1.4131	-0.0001
C	0.0000	-3.5128	0.0002
C	0.0001	3.5128	0.0002
C	2.4451	-3.5102	0.0001
C	2.4451	3.5102	0.0003
C	-2.4451	-3.5102	0.0003
C	-2.4450	3.5102	0.0000
C	3.6544	-2.8137	-0.0001
C	3.6544	2.8136	0.0002
C	-3.6545	-2.8136	0.0002
C	-3.6544	2.8137	-0.0001
C	4.8770	-0.6978	-0.0003
C	4.8771	0.6978	0.0000
C	-4.8771	-0.6977	-0.0001
C	-4.8771	0.6978	-0.0002
H	0.0000	-4.6028	0.0002
H	0.0001	4.6029	0.0004
H	2.4693	-4.5981	0.0002
H	2.4694	4.5981	0.0005
H	-2.4694	-4.5981	0.0004
H	-2.4693	4.5981	0.0000
H	4.5838	-3.3794	0.0000
H	4.5839	3.3793	0.0004
H	-4.5839	-3.3794	0.0003
H	-4.5838	3.3794	-0.0002
H	5.8313	-1.2208	-0.0003
H	5.8313	1.2207	0.0000
H	-5.8313	-1.2206	-0.0001
H	-5.8313	1.2208	-0.0002

Table S3: Geometry of the system **III** in XYZ format (Å).

C	1.2470	0.7040	-0.0009
C	1.2470	-0.7041	-0.0007
C	-1.2470	0.7040	-0.0010
C	-1.2471	-0.7042	-0.0012
C	0.0000	1.3979	-0.0010
C	0.0000	-1.3980	-0.0009
C	2.4797	1.4156	-0.0008
C	2.4798	-1.4156	0.0000
C	-2.4797	1.4155	-0.0004
C	-2.4797	-1.4157	-0.0015
C	0.0001	2.8084	-0.0013
C	0.0000	-2.8085	-0.0007
C	3.7271	0.7078	-0.0003
C	3.7271	-0.7078	0.0006
C	-3.7270	0.7077	-0.0001
C	-3.7270	-0.7079	-0.0015
C	2.4065	2.8256	-0.0010
C	2.4066	-2.8257	0.0001
C	-2.4064	2.8256	-0.0007
C	-2.4066	-2.8257	-0.0010
C	1.1976	3.5096	-0.0014
C	-1.1975	3.5095	-0.0011
C	1.1976	-3.5097	-0.0002
C	-1.1975	-3.5097	-0.0005
C	4.9896	1.3690	-0.0007
C	4.9898	-1.3690	0.0018
C	-4.9896	1.3690	0.0018
C	-4.9897	-1.3690	-0.0028
C	6.2236	0.6985	0.0005
C	6.2238	-0.6984	0.0019
C	-6.2237	0.6985	0.0019
C	-6.2238	-0.6984	-0.0008
C	7.4495	1.3891	0.0000
C	7.4498	-1.3889	0.0029
C	-7.4496	1.3892	0.0047
C	-7.4498	-1.3888	-0.0014
C	8.6595	0.6954	0.0004
C	8.6595	-0.6950	0.0019
C	-8.6595	0.6954	0.0043
C	-8.6596	-0.6949	0.0012
H	3.2969	3.4475	-0.0009
H	3.2969	-3.4475	0.0004
H	-3.2968	3.4475	-0.0007
H	-3.2969	-3.4477	-0.0008
H	1.2130	4.5975	-0.0015
H	-1.2129	4.5975	-0.0012
H	1.2131	-4.5976	0.0000
H	-1.2129	-4.5976	-0.0001
H	5.0376	2.4561	-0.0018
H	5.0378	-2.4562	0.0027
H	-5.0375	2.4562	0.0037
H	-5.0377	-2.4561	-0.0048
H	7.4699	2.4770	-0.0012
H	7.4703	-2.4768	0.0040
H	-7.4700	2.4771	0.0071
H	-7.4704	-2.4767	-0.0035
H	9.5987	1.2405	-0.0002
H	9.5988	-1.2399	0.0024
H	-9.5987	1.2405	0.0063
H	-9.5989	-1.2398	0.0009

Table S4: Geometry of the system **IV** (peripentacene) in XYZ format (Å).

C	14.13369621	24.09738288	7.41419320
C	15.52860709	24.11671801	7.41418866
C	16.27758770	22.93689028	7.41418564
C	17.74444497	22.93682197	7.41418441
C	18.49325442	24.11677618	7.41418346
C	19.88806712	24.09763674	7.41418100
C	20.57223729	22.90243977	7.41418259
C	19.86834535	21.67999544	7.41418577
C	20.54741789	20.44587349	7.41419006
C	19.86832179	19.22959736	7.41419500
C	20.54839156	17.99915476	7.41420924
C	19.86812585	16.76892065	7.41421238
C	20.54707494	15.55233190	7.41419817
C	19.86777884	14.31850274	7.41419387
C	18.43606199	14.30213245	7.41419137
C	20.57149434	13.09598297	7.41419271
C	19.88719903	11.90094885	7.41418828
C	18.49228589	11.88199236	7.41418659
C	17.74374134	13.06205824	7.41419023
C	16.27683440	13.06230394	7.41418831
C	15.52769994	11.88255792	7.41418569
C	14.13277939	11.90212127	7.41418335
C	13.44907613	13.09744558	7.41418412
C	14.15343034	14.31959321	7.41418731
C	15.58516328	14.30264567	7.41418791
C	16.29582174	15.54366728	7.41419103
C	17.72594899	15.54346419	7.41419284
C	18.42404082	16.76403810	7.41419318
C	17.72016424	17.99945327	7.41419368
C	18.42423720	19.23472914	7.41419295
C	17.72628478	20.45532510	7.41418692
C	16.29617552	20.45553751	7.41418803
C	18.43657439	21.69663318	7.41418555
C	15.58570198	21.69660930	7.41418551
C	14.15398921	21.67992894	7.41418556
C	13.44979816	22.90219735	7.41419266
C	13.47500551	20.44609659	7.41418861
C	14.15433553	19.22994299	7.41419101
C	15.59828148	19.23486604	7.41419063
C	15.59809424	16.76441229	7.41419457
C	16.30229099	17.99958278	7.41419044
C	14.15411872	16.76953807	7.41418855
C	13.47400409	17.99979206	7.41417884
C	13.47461040	15.55350292	7.41418763
H	13.59028745	25.03198133	7.41419659
H	16.02125699	25.07598052	7.41418836
H	18.00014455	25.07580095	7.41418311
H	20.43145300	25.03222835	7.41417890
H	21.65369521	22.88888590	7.41418195
H	21.62972366	20.44627211	7.41419173
H	21.62938053	15.55201132	7.41420002
H	21.65294363	13.10947409	7.41419432
H	20.43032253	10.96620056	7.41418719
H	17.99917719	10.92296453	7.41418390
H	16.02022255	10.92323413	7.41418572
H	13.58913150	10.96767614	7.41418106
H	12.36763995	13.11178004	7.41418273
H	12.36836644	22.88799347	7.41419633
H	12.39272858	20.44587882	7.41418770
H	12.39183423	17.99987347	7.41417405
H	12.39233706	15.55384728	7.41418436
H	21.63056990	17.99921526	7.41421445

Table S5: Geometry of the system **V** in XYZ format (Å).

C	-5.6025	-0.0066	0.0009
H	-6.6825	-0.0079	0.0020
C	-4.9125	1.1916	-0.0006
H	-5.4544	2.1258	-0.0010
C	-3.5092	1.1956	-0.0009
C	-2.8518	2.4343	-0.0019
H	-3.4258	3.3491	-0.0024
C	-1.4604	2.4870	-0.0016
C	-0.6970	3.6621	-0.0015
H	-1.1886	4.6237	-0.0020
C	0.6929	3.5934	-0.0001
C	1.4606	4.7782	0.0008
H	0.9820	5.7464	0.0011
C	2.8214	4.6725	0.0017
H	3.4204	5.5712	0.0028
N	3.4255	3.4908	0.0016
C	2.7423	2.3440	0.0008
N	1.3691	2.3902	-0.0001
C	0.6577	1.2137	-0.0008
C	1.3508	0.0016	-0.0007
C	2.8214	0.0034	0.0001
N	3.4286	-1.1818	0.0002
C	2.7481	-2.3373	-0.0002
N	1.3750	-2.3870	-0.0006
C	0.6607	-1.2123	-0.0009
C	-0.7338	-1.2048	-0.0010
C	-1.4500	-0.0018	-0.0007
N	-2.8257	-0.0034	0.0000
C	-3.5064	-1.2041	-0.0001
C	-2.8459	-2.4412	-0.0010
H	-3.4178	-3.3574	-0.0012
C	-1.4544	-2.4908	-0.0010
C	-0.6879	-3.6638	-0.0006
H	-1.1772	-4.6267	-0.0012
C	0.7018	-3.5919	-0.0002
C	1.4726	-4.7746	0.0003
H	0.9964	-5.7440	0.0001
C	2.8331	-4.6655	0.0007
H	3.4343	-5.5627	0.0008
N	3.4343	-3.4823	0.0001
C	-4.9097	-1.2032	0.0068
H	-5.4494	-2.1387	0.0071
C	-0.7368	1.2028	-0.0011
N	3.4257	1.1902	0.0008

References

(S1) Lee, C.; Yang, W.; Parr, R. G. Development of the Colle-Salvetti Correlation-Energy Formula into a Functional of the Electron Density. *Phys. Rev. B* **1988**, *37*, 785–789.

(S2) Becke, A. D. Density-Functional Exchange-Energy Approximation with Correct Asymptotic Behavior. *Phys. Rev. A* **1988**, *38*, 3098–3100.

(S3) Dunning, T. H. Gaussian Basis Sets for use in Correlated Molecular Calculations. I. The Atoms Boron Through Neon and Hydrogen. *J. Chem. Phys.* **1989**, *90*, 1007–1023.

(S4) Pipek, J.; Mezey, P. G. A fast intrinsic localization procedure applicable for ab initio and semiempirical linear combination of atomic orbital wave functions. *The Journal of Chemical Physics* **1989**, *90*, 49164926.

(S5) Barcza, G.; Legeza, O.; Marti, K. H.; Reiher, M. Quantum-information analysis of electronic states of different molecular structures. *Phys. Rev. A* **2011**, *83*, 012508.

(S6) Olivares-Amaya, R.; Hu, W.; Nakatani, N.; Sharma, S.; Yang, J.; Chan, G. K.-L. The Ab-Initio Density Matrix Renormalization Group in Practice. *J. Chem. Phys.* **2015**, *142*, 034102.

(S7) Legeza, O.; Sólyom, J. Optimizing the Density-Matrix Renormalization Group Method Using Quantum Information Entropy. *Phys. Rev. B* **2003**, *68*, 195116.

(S8) Hinton, G. E.; Srivastava, N.; Krizhevsky, A.; Sutskever, I.; Salakhutdinov, R. R. Improving Neural Networks by Preventing Co-Adaptation of Feature Detectors. arXiv:1207.0580, 2012.

(S9) Srivastava, N.; Hinton, G.; Krizhevsky, A.; Sutskever, I.; Salakhutdinov, R. Dropout: A Simple Way to Prevent Neural Networks from Overfitting. *J. Mach. Learn. Res.* **2014**, *15*, 1929 – 1958.

(S10) An end-to-end open source machine learning platform. *https://www.tensorflow.org/*

(S11) PyTorch Geometric. *https://pytorch-geometric.readthedocs.io/*

(S12) Sánchez-Grande, A.; Urgel, J. I.; Veis, L.; Edalatmanesh, S.; Santos, J.; Lauwaet, K.; Mutombo, P.; Gallego, J. M.; Brabec, J.; Beran, P.; Nachtigallová, D.; Miranda, R.; Martín, N.; Jelínek, P.; Écija, D. Unravelling the Open-Shell Character of Peripentacene on Au(111). *J. Phys. Chem. Lett.* **2020**, *12*, 330–336.

(S13) https://github.com/golubp/QC_DMRG_pred, accessed 2024-12-06.
