# Supplementary figures and images for: Quantum Chemical Density Matrix Renormalization Group Method Boosted by Machine Learning
Source: J Phys Chem Lett. 2025 Mar 24;16(13):3295–301. doi: 10.1021/acs.jpclett.5c00207 (PMC11973911; doi:10.1021/acs.jpclett.5c00207)

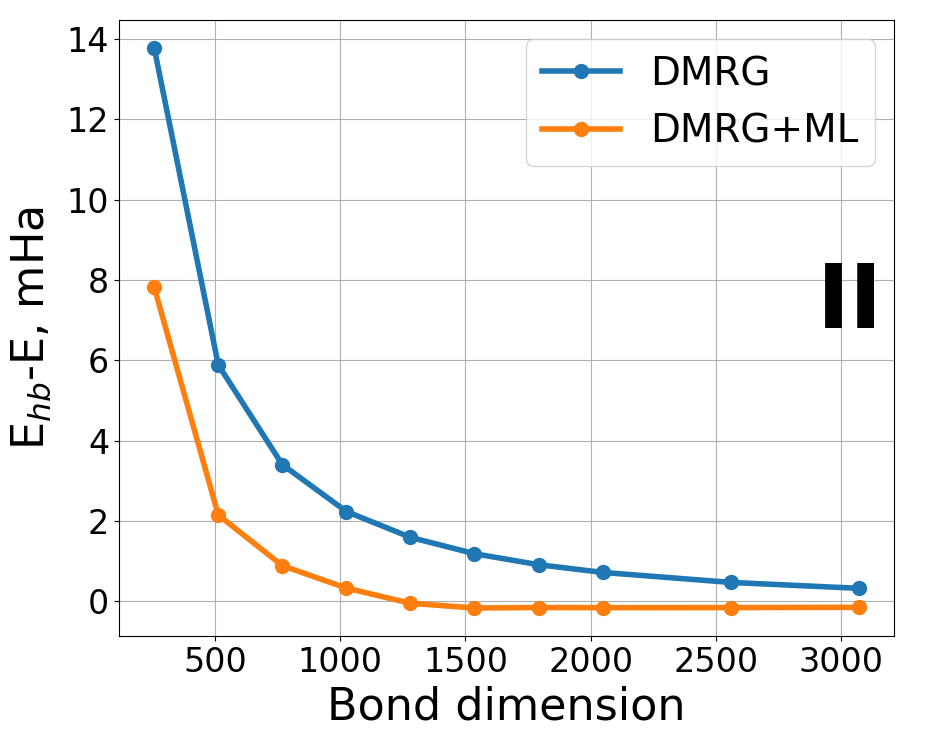

Supplement: Supplementary file 2 — jz5c00207_si_002.zip [file jz5c00207_si_002.zip › Supporting/Fig_S1a.png]

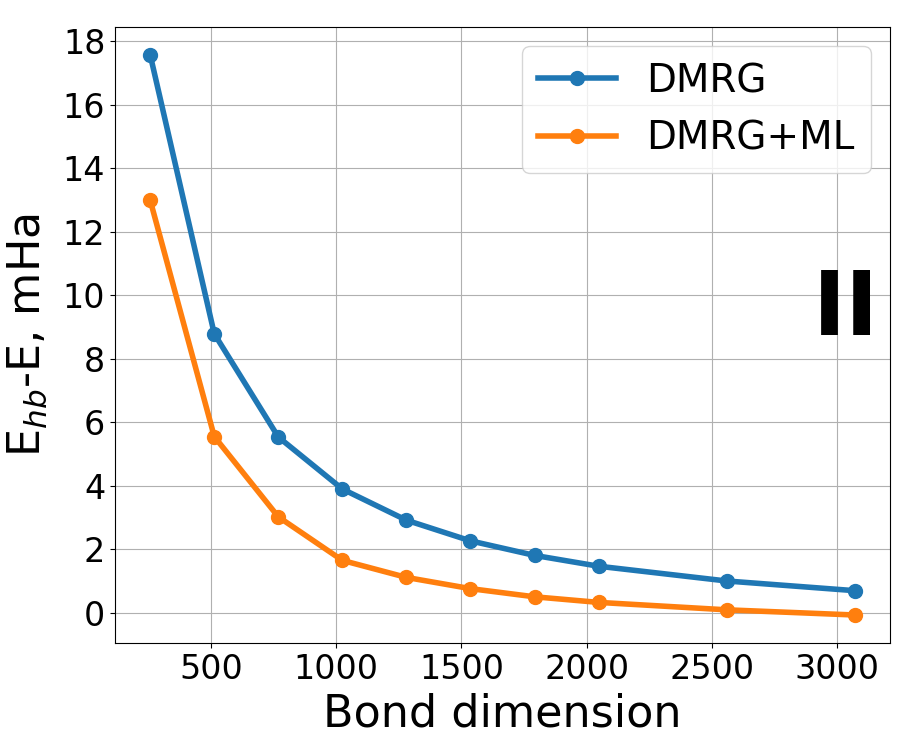

Supplement: Supplementary file 2 — jz5c00207_si_002.zip [file jz5c00207_si_002.zip › Supporting/Fig_S1b.png]

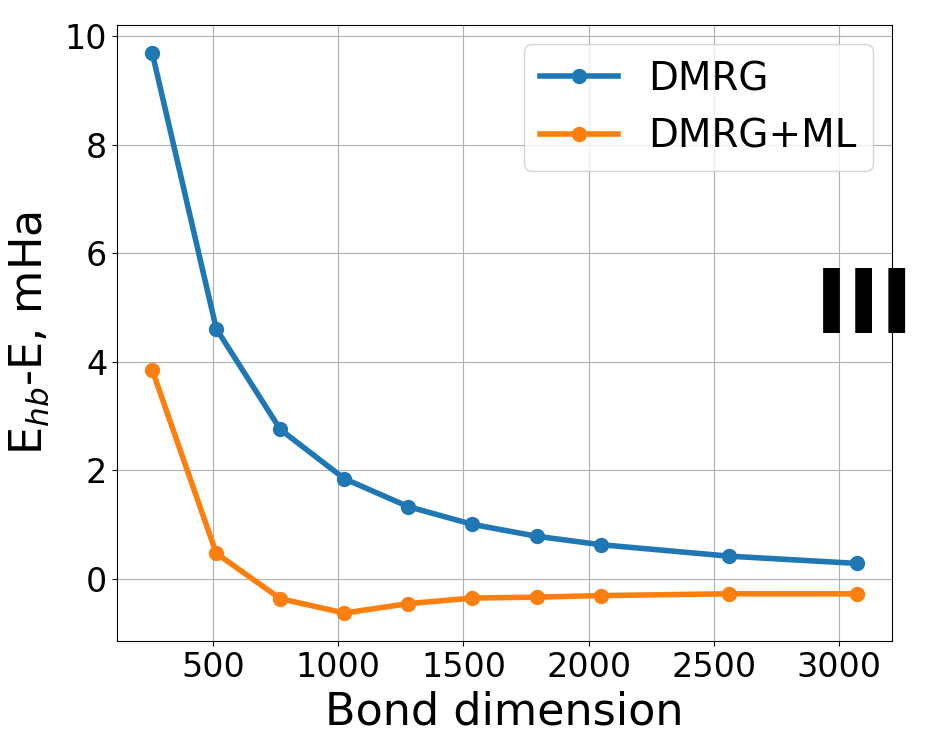

Supplement: Supplementary file 2 — jz5c00207_si_002.zip [file jz5c00207_si_002.zip › Supporting/Fig_S1c.png]

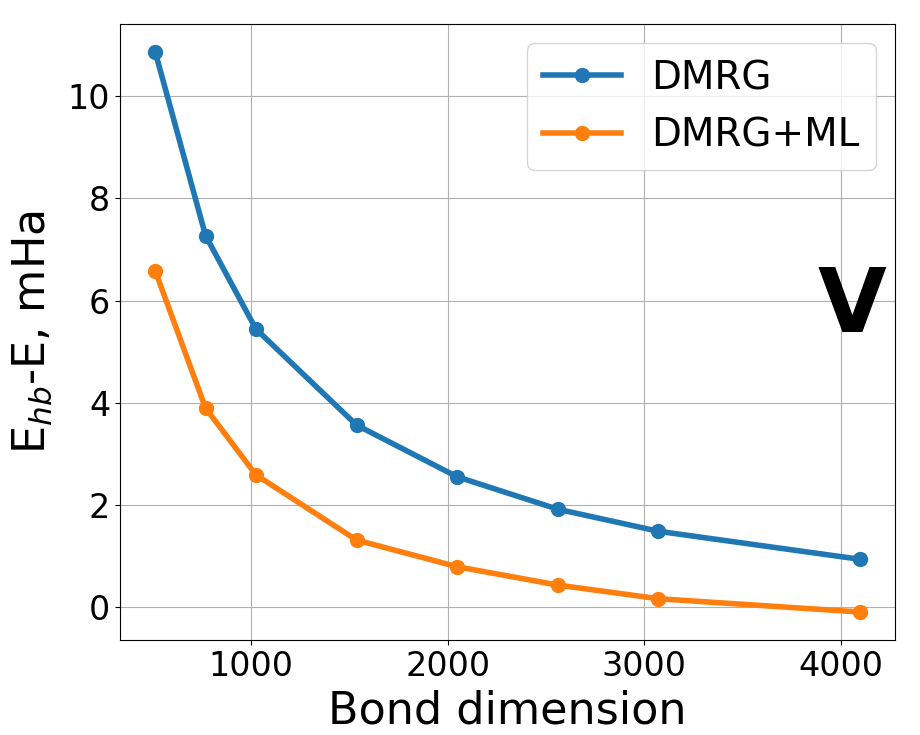

Supplement: Supplementary file 2 — jz5c00207_si_002.zip [file jz5c00207_si_002.zip › Supporting/Fig_S1d.png]

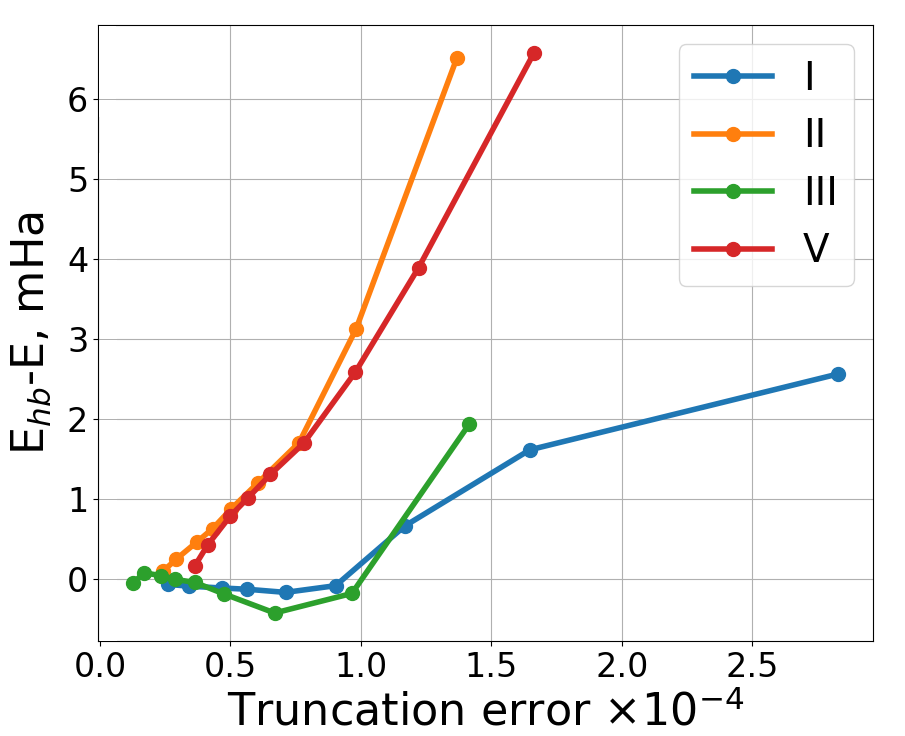

Supplement: Supplementary file 2 — jz5c00207_si_002.zip [file jz5c00207_si_002.zip › Supporting/Fig_S1e.png]

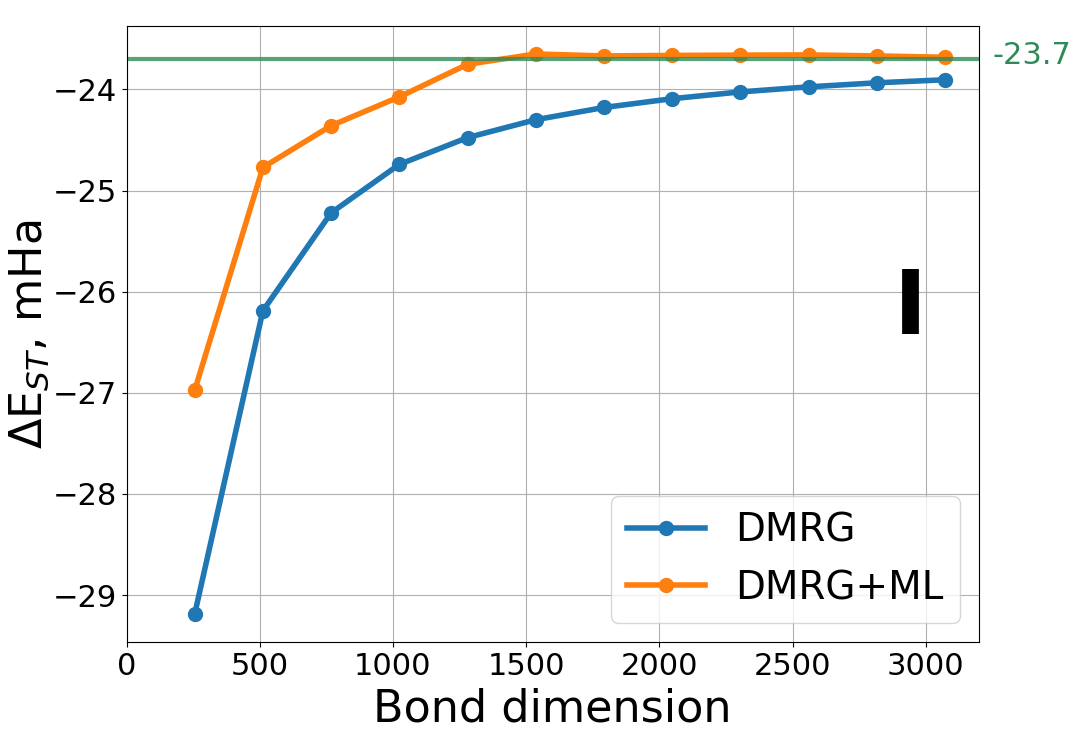

Supplement: Supplementary file 2 — jz5c00207_si_002.zip [file jz5c00207_si_002.zip › Supporting/Fig_S2.png]

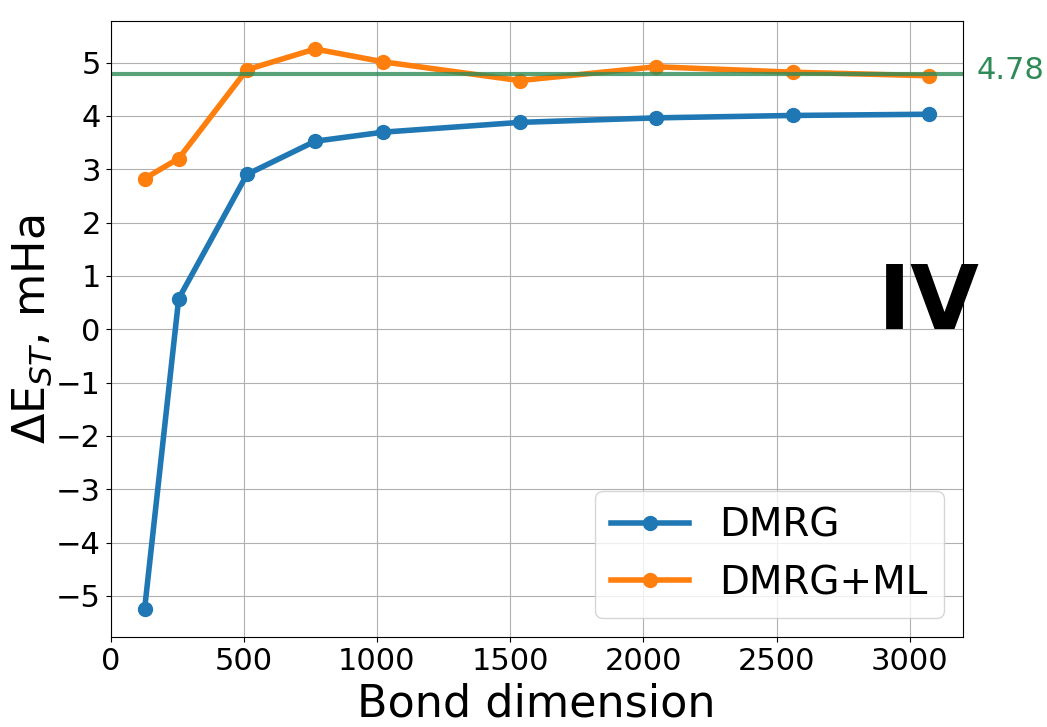

Supplement: Supplementary file 2 — jz5c00207_si_002.zip [file jz5c00207_si_002.zip › Supporting/Fig_S3.png]

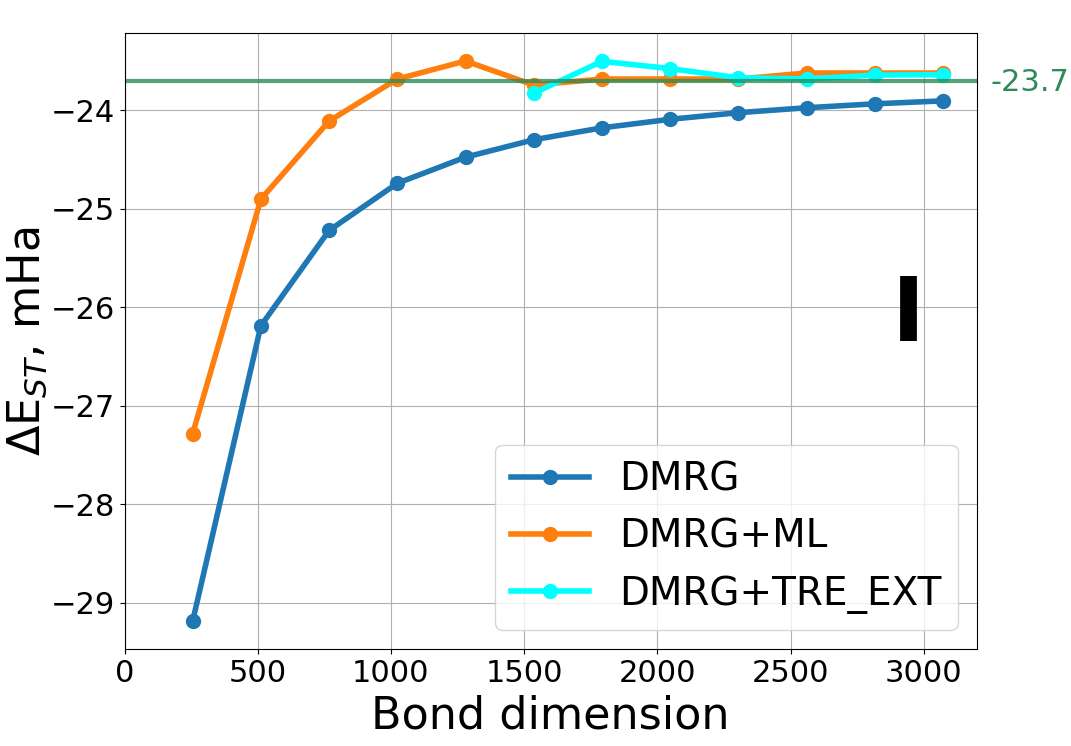

Supplement: Supplementary file 2 — jz5c00207_si_002.zip [file jz5c00207_si_002.zip › Supporting/Fig_S4.png]

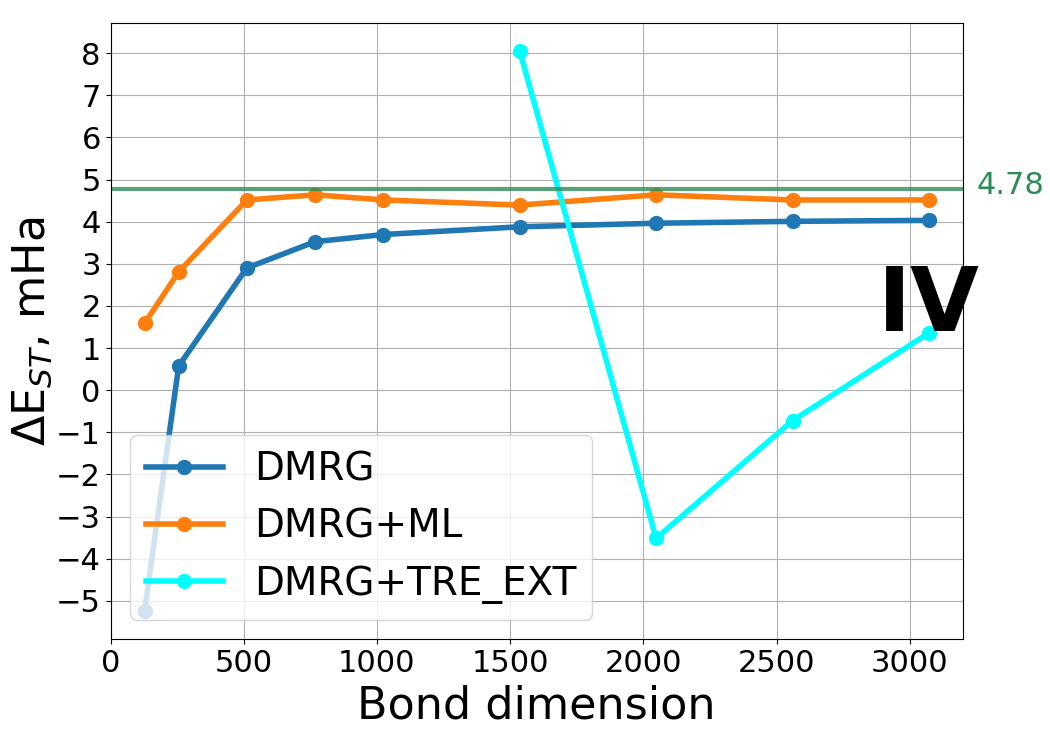

Supplement: Supplementary file 2 — jz5c00207_si_002.zip [file jz5c00207_si_002.zip › Supporting/Fig_S5.png]
